# Metabolic Cardiovascular Renal Disease (Met‐CVRD): A New Nomenclature

**DOI:** 10.1002/dmrr.70083

**Published:** 2025-09-01

**Authors:** Paolo Pozzilli, Maria Vittoria Messina, Michael Roden

**Affiliations:** ^1^ Fondazione Policlinico Universitario Campus Bio‐Medico Rome Italy; ^2^ The Blizard Institute St. Bartholomew's and the London School of Medicine London UK; ^3^ Department of Endocrinology and Diabetology Medical Faculty and University Hospital Heinrich Heine University Düsseldorf Germany; ^4^ Institute for Clinical Diabetology German Diabetes Center Leibniz Institute for Diabetes Research at Heinrich Heine University Düsseldorf Germany; ^5^ German Center for Diabetes Research (DZD e.V.) München Germany

**Keywords:** cardiovascular renal disease, metabolic syndrome, type 2 diabetes

## Abstract

Cardiovascular Renal Disease (CVRD) has been introduced as a syndrome describing the coexistence of certain common diseases. However, this terminology misses the role of metabolic abnormalities not only as relevant comorbidities, but even more as key causal factors. Thus, the word ‘metabolic’ should come first to define this syndrome as its joint underlying pathogenesis. Although, CVRD and type 2 diabetes (T2D) have historically been treated as coexisting but separate conditions, growing evidence underscores a bidirectional and metabolically driven relationship between the heart and kidneys, mediated by upstream processes. These comprise insulin resistance, ectopic lipid deposition, mitochondrial abnormalities, dyslipidaemia and chronic low‐grade inflammation, typical of T2D and the metabolic syndrome. These metabolic disturbances begin silently in early adulthood, well before traditional clinical markers can signal CVRD onset. Here, we introduce the new terminology of Metabolic Cardiovascular Renal Disease (Met‐CVRD), to indicate that the word ‘Metabolic’ represents its major pathogenic factor. We then discuss then the necessity of prioritising early at‐risk individual identification and prompt intervention with cardiorenal‐protective therapies like glucagon‐like peptide‐1 (GLP‐1) receptor agonists and sodium–glucose cotransporter‐2 (SGLT2) inhibitors. Met‐CVRD provides a cohesive and proactive strategy to halt the advancement of cardiorenal disease across several systems by transcending organ‐specific frameworks.

## 1 The Cardiorenal Axis: Chronic Kidney Disease in the Context of Cardiovascular Risk

Cardiovascular renal disease (CVRD) refers to the interrelated and often concurrent presence of cardiovascular disease (CVD) and chronic kidney disease (CKD). This term reflects the bidirectional relationship between the heart and kidneys. Indeed, as early as the late 1990s and early 2000s, epidemiological studies began to show that CKD and CVD often co‐exist, even in individuals with only mild renal impairment. Most recent studies accounting for this cardiorenal association are quoted [1]. However, previous models did not fully account for the key upstream metabolic dysfunctions—such as insulin resistance, chronic low‐grade inflammation, and dyslipidaemia—that precede and accelerate both cardiac and renal outcomes.

## 2 Metabolic Cardiovascular Renal Disease (Met‐CVRD): Uncovering the Metabolic Origins of Cardiorenal Dysfunction

**FIGURE 1:**
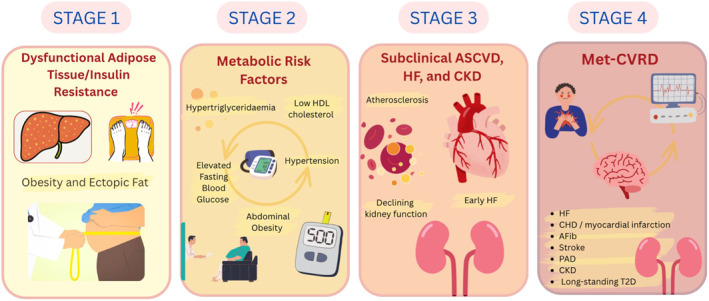
The central role of metabolic dysfunction in the pathogenesis of CVKD. This diagram illustrates the progressive development of Met‐CVRD in different stages, starting from early metabolic dysfunction to advanced clinical outcomes. Stage 1: Characterized by excess or dysfunctional adipose tissue, leading to metabolic disturbances such as overweight/obesity, impaired glucose tolerance, and fatty liver (ectopic fat). Stage 2: Involves key metabolic risk factors including elevated fasting blood glucose, hypertension, hypertriglyceridaemia, low HDL cholesterol, and abdominal obesity, all contributing to the development of cardiorenal damage. Stage 3: Represents the subclinical stage of atherosclerotic cardiovascular disease (ASCVD), heart failure (HF), and CKD, marked by early atherosclerosis, declining kidney function, and early signs of HF. Stage 4: Reflects overt Met‐CVRD associated with advanced CKD, Coronary Heart Disease (CHD), myocardial infarction, HF, Stroke, Peripheral Artery Disease (PAD), T2D, and Atrial Fibrillation (Afib).

Introduction of the term Cardiovascular‐Kidney‐Metabolic (CKM) syndrome was indeed a step towards better description of this complex coexistence of chronic diseases, and it underscores the need for multidisciplinary prevention [2]. The structure of our novel staging model was inspired by the CKM syndrome framework (Ndumele et al., Circulation, 2023); however, we intentionally reordered the terminology to emphasize the central role of metabolic dysfunction in disease progression. Specifically, we propose to move the term ‘Metabolic’ to the front, resulting ‘Metabolic cardiovascular renal disease (Met‐CVRD)’ to underline the primary role of the metabolic dysfunction within the interconnections between cardiovascular and renal disorders. Unlike previous models that viewed cardiovascular and kidney diseases as parallel but distinct entities, the Met‐CVRD framework acknowledges the complex metabolic alterations as the common upstream driver. These alterations comprise, mostly starting with adipose tissue dysfunction and local insulin resistance, abnormal energy metabolism with ectopic lipid deposition and dyslipidaemia and ultimately chronic low‐grade inflammation, which initiate and accelerate both cardiac and renal impairments (see Figure 1).

Notably, the origins of this so‐called metabolic dysfunction are neither sudden nor exclusive to older age but can start to be detectable as early as the third or fourth decade of life [3]. Indeed, when evaluated using standard or advanced clinical biomarkers, such as estimated glomerular filtration rate (eGFR), albuminuria or cardiovascular imaging throughout these early stages, cardiorenal function frequently stays within physiological range, potentially obscuring the underlying pathophysiological processes. Nevertheless, the metabolic groundwork for long‐term damage is already being laid [4]. As a matter of fact, early metabolic indicators—including increased waist circumference [5], indices of insulin resistance and circulating biomarkers including adipokines and haemoglobin A1c (HbA1c),—have been shown to precede the clinical manifestation of CVRD by several years [6]. Their presence is now acknowledged as a strong prognostic marker for future cardiorenal complications, even in individuals who are otherwise asymptomatic.

To understand the origin of these early pathophysiological changes, it is essential to consider the age‐related physiological changes that progressively undermine metabolic homeostasis. An early clinically detectable feature, that is impaired glucose metabolism, is attributable to the gradual decline in insulin sensitivity, mitochondrial efficiency, and vascular elasticity. Changes in body composition, namely an increase in visceral adipose tissue (abdominal obesity) and a decrease in skeletal muscle mass (sarcopenia), rising progressively with increasing age and obesity, further aggravate this process [7]. Indeed, abnormal mitochondrial function, increasingly prevalent with age and obesity, compromises cellular energy production and is closely linked to insulin resistance and lipotoxicity [6]. Furthermore, sustained hyperglycaemia perpetuates the cycle through glucotoxicity, leading to β‐cell dysfunction, and through the accumulation of advanced glycation end‐products (AGEs) and oxidative stress [8], which in turn damage vascular and organ tissues.

This long subclinical phase, invisible to standard diagnostics but biologically active, is precisely where the Met‐CVRD model seeks to intervene. By identifying individuals at risk much earlier, clinicians can act before the ‘point of no return’, when structural and functional decline in the heart or kidneys becomes increasingly harder to reverse. Recent efforts of subtyping persons without or with early T2D revealed that those with severe insulin resistance, visceral and ectopic lipid storage were at the highest risk of developing both CVD and CKD [9, 10]. The goal is therefore not simply to manage disease, but to prevent its clinical emergence as early as possible. This is where targeted interventions, such as early use of cardiorenal‐protective agents (e.g., SGLT2 inhibitors, GLP‐1 receptor agonists), gain relevance—not just as glucose‐lowering agents, but as tools for disease modification with benefits extending across organ systems [11].

Supporting this approach, a large retrospective analysis by Birkeland et al. involving more than 1.1 million persons with T2D provides compelling data to support our change in nomenclature. Over an average 4.5‐year follow‐up, 18% of people with T2D who were initially CVRD‐free, developed a CVRD condition, with HF or CKD accounting for 60% of first manifestations [12]. These findings reinforce the idea that HF and CKD are not just complications—but represent the ultimate outcomes in T2D as the consequence of metabolic dysregulation.

A recent study by Sattar et al. concurrently demonstrates the complexity and multidimensionality of Met‐CVRD. Their analysis showed that cardiovascular events, such as heart failure, stroke, and coronary artery disease were independently predicted by HbA1c, blood pressure (both systolic and diastolic), LDL‐C, triglycerides, and BMI. Interestingly, HbA1c was most significantly linked to atherosclerotic events, but BMI was especially predictive of heart failure [13]. *These findings emphasize the need for comprehensive metabolic control*, not just of glucose, but also of body weight, lipids, and blood pressure, as a cornerstone of cardiovascular and renal prevention.

Taken together, growing evidence validates the Met‐CVRD model as a practical and clinically feasible framework. While challenging some traditional approaches to common chronic diseases, this model urges a shift towards early, integrated, and multi‐system screening and prevention. This model also redefines the management of T2D, not as a condition to be tolerated until complications arise, but as the key metabolic tipping point where early intervention can radically alter the trajectory of health across the heart, the kidneys, and beyond.

## Author Contributions

Paolo Pozzilli, Maria Vittoria Messina and Michael Roden have equally contributed to the conceptualization, writing–original draft, review and editing, approval of the final version submitted for publication.

## Disclosure

The authors have nothing to report.

## Ethics Statement

This is a review article on a topic of general interest that does not require any approval by Ethical Committee.

## Conflicts of Interest

The authors declare no conflicts of interest.

## Data Availability

Data sharing is not applicable to this article as no new data were created or analyzed in this study.
